# RE: “Narrative review of lifestyle interventions in breast cancer survivors: current evidence and future directions”

**DOI:** 10.1093/jncics/pkae125

**Published:** 2024-12-20

**Authors:** Alain Braillon

**Affiliations:** 80000 Amiens, France

Gabel and colleagues must be commended for having reviewed behavioral interventions in breast cancer survivors because weight gain, a frequent issue, increases the risk of breast cancer recurrence and also obesity-related comorbidities.^1^ However, comments are warranted.

First, the issue has not attracted the concern it deserves. Quantitatively, among 30 interventions that combined diet and physical activity among breast cancer survivors, only 2 are randomized controlled trials with more than 100 participants in each group. Qualitatively, concluding that “data on cardiometabolic benefit is unclear and more studies are needed to elucidate the long-term health benefits among breast cancer survivors”^1^ seems a euphemism for no evidence, even on surrogate, to identify an intervention allowing to expect benefits on relevant clinical outcomes.

Second, I am concerned by the use of the slogan “one in eight females will be diagnosed with breast cancer in their lifetime.”^1^ On one hand, the statistic is that 1 in 8 women *who survive to age 85* (my emphasis) will develop some form of breast cancer, but this is also a 7 in 8 chance she will never have the disease. In my opinion and as a clinician, reassurance is preferable to fearmongering, even more as women largely overestimate their probability of dying of breast cancer.^2^ On the other hand, the use of this slogan masks the importance of preventable risk factors; weight management in survivors is the tip of the iceberg. Is it acceptable that breast cancer screening flies in the face of basic public health principles for prevention? Why is screening advocates’ aim to increase the use of screening mammography rather than to develop optimal strategies? Can’t precision medicine, a concept developed in 1998 when the BCR-ABL rearrangement in chronic myeloid leukemia was successfully targeted by imatinib, be implemented for screening? The shared decision-making process before mammography should take into account personal risk, even more because several risk factors are preventable: not being physically active, being overweight, taking hormones, drinking alcohol, smoking, and so on.^3^ Why do organizations promoting screening fail to provide adequate decision aids allowing women to make informed decisions about the benefit to harm ratio of screening taking into account lifestyle for personal risk?^4^ For example, the Breast Cancer Risk Assessment Tool (The Gail Model) from the National Institute of Health ignores preventable risk factors.^5^

Screening without risk-stratified strategy, as lack of funding for quality assurance programs, is a breach of trust wasting major resources without guaranteeing effectiveness and safety. The promotion of a healthy lifestyle should be a mandatory prerequisite of screening programs.

## Data Availability

Not applicable.
